# Potential Benefits of Black Chokeberry (*Aronia melanocarpa*) Fruits and Their Constituents in Improving Human Health

**DOI:** 10.3390/molecules27227823

**Published:** 2022-11-13

**Authors:** Yulin Ren, Tyler Frank, Gunnar Meyer, Jizhou Lei, Jessica R. Grebenc, Ryan Slaughter, Yu G. Gao, A. Douglas Kinghorn

**Affiliations:** 1 Division of Medicinal Chemistry and Pharmacognosy, College of Pharmacy, The Ohio State University, Columbus, OH 43210, USA; 2 OSU South Centers, The Ohio State University, Columbus, OH 43210, USA; 3 Department of Horticulture and Crop Science, College of Food, Agricultural, and Environmental Sciences, The Ohio State University, Columbus, OH 43210, USA

**Keywords:** black chokeberry, *Aronia melanocarpa*, phenolic constituents, antioxidants, anti-infectives, antitumor effects, human health

## Abstract

Aronia berry (black chokeberry) is a shrub native to North America, of which the fresh fruits are used in the food industry to produce different types of dietary products. The fruits of *Aronia melanocarpa* (Aronia berries) have been found to show multiple bioactivities potentially beneficial to human health, including antidiabetic, anti-infective, antineoplastic, antiobesity, and antioxidant activities, as well as heart-, liver-, and neuroprotective effects. Thus far, phenolic compounds, such as anthocyanins, cyanidins, phenolic acids, proanthocyanidins, triterpenoids, and their analogues have been identified as the major active components of Aronia berries. These natural products possess potent antioxidant activity, which contributes to the majority of the other bioactivities observed for Aronia berries. The chemical components and the potential pharmaceutical or health-promoting effects of Aronia berries have been summarized previously. The present review article focuses on the molecular targets of extracts of Aronia berries and the examples of promising lead compounds isolated from these berries, including cyanidin-3-*O*-galactoside, chlorogenic acid, quercetin, and ursolic acid. In addition, presented herein are clinical trial investigations for Aronia berries and their major components, including cancer clinical trials for chlorogenic acid and COVID-19 trial studies for quercetin. Additionally, the possible development of Aronia berries and their secondary metabolites as potential therapeutic agents is discussed. It is hoped that this contribution will help stimulate future investigations on Aronia berries for the continual improvement of human health.

## 1. Introduction

**Figure 1:**
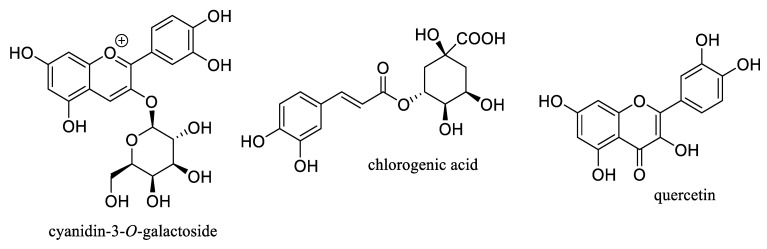
Structures of selected major phenolic compounds isolated from Aronia berries.

Aronia berry or black chokeberry, *Aronia melanocarpa* (Michx.) Elliott (Rosaceae), is a shrub native to North America, of which the fresh fruits are not typically consumed directly, owing to their bitter taste. However, these berries are used in the food and beverage industry for producing juices, syrups, jams, fruit teas and wine, and are also utilized in dietary supplements [1,2]. Phytochemical investigations have shown that the fruits of *Aronia melanocarpa* (Aronia berries) are an abundant source of phenolic compounds, including procyanidins, anthocyanins, phenolic acids, and their analogues [2,3]. These berries also produce several novel compounds containing a fused flavanol-coumarin-phenol unit and showing hydroxyl radical scavenging and quinone reductase-inducing activities [4]. The content of the major bioactive components of Aronia berries is relatively high, from 10 mg to 5500 mg per 100 g of the dried fruits, including procyanidins, cyanidin-3-*O*-galactoside, chlorogenic acid, and quercetin (Figure 1). Of these, the content of procyanidins may be greater than 5% of the dried fruits. The high concentrations of these bioactive compounds not only contribute to the biological effects observed for Aronia fruits but also present the potential for the discovery of useful therapeutic agents from this plant part [1,2,3].

The phenolic compounds present potent antioxidative activities that contribute to the health-promoting activities of Aronia berries. These include antidiabetic, anti-infective, antimutagenic, and cytotoxic activities and cardio-, gastro-, hepato-, and radio-protective and immunomodulatory effects [5]. Thus, consumption of Aronia berries could be supportive of the prevention of some chronic diseases, including metabolic disorder [6], and may reduce the toxic effects of some xenobiotics on human health [7].

Research progress on Aronia berries has been summarized in several review articles published recently [1,2,3,6,7]. However, these previous reviews focused mainly on the chemical components, product preparation, and pharmaceutical effects of the extracts or bioactive component-enriched extracts of Aronia berries. The mechanisms of action and molecular targets of the active compounds and their clinical trial investigations have not been well discussed thus far. In addition, the bioactivities of triterpenoid components and the anti-COVID-19 potential of ursolic acid and quercetin, which are the major components of Aronia berries, have not been summarized in any previous reviews on these berries. Thus, the present review focuses on the bioactive components of Aronia berries and their mechanisms of action, with clinical trial studies being highlighted. It is hoped to provide some important information for the development of useful agents from Aronia berries to improve human health.

## 2. Antioxidant Activity of Aronia Berries

The antioxidant activity of Aronia berries and their major phenolic constituents has been well documented recently. These natural products have been found to inhibit the activity of several types of radicals through different mechanisms of action to contribute to other bioactivities [8,9]. The extracts and several phenolic compounds of Aronia berries showed radical scavenging activity when tested by a 1,1-diphenyl-2-picrylhydrazyl (DPPH) assay, and they also inhibited 15-lipoxygenase and xanthine oxidase, which are peroxidative and prooxidative enzymes, respectively, the sources of reactive oxygen species (ROS) in vascular cells [8]. Previously, several small phenolic compounds isolated from Aronia berries in our research group were found to show potent antioxidant activity when tested in a hydroxyl radical-scavenging assay, and the catechol group in these compounds seems to play an important role in mediating such a property [10,11]. The antioxidant activity of Aronia berries has also been investigated in a clinical trial on 11 healthy human volunteer subjects who drank daily 250 mL of the juice of Aronia berries (Aronia juice) for three weeks. The serum antioxidant capacity of the participants was increased significantly when tested by a spectrophotometric method, using DPPH stable radical cations [12].

However, a subsequent clinical trial investigation indicated that consumption of Aronia berries did not change the biomarkers of oxidative stress and the total antioxidant activity measured in both plasma and urine of the participants when a 12-week, randomized, placebo-controlled trial was conducted on 49 healthy adult former smokers who consumed daily 500 mg of the ethanolic extract of Aronia berries [13]. Similarly, Aronia juice supplementation was not found to affect the parameters measured in the participants when the diet of 12 young male athletes was supplemented daily with 200 mL of Aronia juice (equivalent to 330.6 mg of anthocyanins) for seven weeks [14]. These results indicate that the antioxidant capacity of Aronia berries may not have been sufficient in these studies, and the dose of their phenolic constituents could be important in mediating the resultant activity. Thus, future clinical trials could focus on both optimizing the active dose and studying the toxic effects of Aronia berries [15].

Antioxidants are important in supporting human health, owing to their ability to inhibit free radicals that damage normal cells. Thus, antioxidant effects could be of value in alleviating other conditions that result from oxidative stress, including cancer, infection, heart disease, and diabetes. In this regard, the abundant phenolic compounds and other natural products in Aronia berries that exhibit potent antioxidant activity could be supportive in improving human health [8].

## 3. Potential Antitumor Activity of Aronia Berries

Oxidative stress is also found in various cancer cells, and antioxidants have been regarded as having potential value in cancer chemotherapy. Antioxidant extracts, constituents, or their semi-synthetic derivatives of Aronia berries have been well documented for their potential therapeutic effects on different cancer cells, including human breast, cervical, colon, glioblastoma, liver, and lung cancer and leukemia cells [1,2,3]. For example, the antioxidant activity of Aronia berries was found to be correlated with the total procyanidin and anthocyanin content, and the cyanidin glycosides present inhibited HeLa human cervical cancer cell proliferation and increased the generation of reactive oxygen species (ROS) in these cancer cells [16]. When tested in a further in vitro assay, the phenolic components present in Aronia berries were found to exhibit potent antioxidant activity and cytotoxicity toward HepG2 human liver cancer cells [17].

Aronia berries show promising activity toward human colon cancer cells. Commercially available extracts of the fruits of red [*Aronia arbutifolia* (L.) Pers.], purple [*Aronia prunifolia* (Marshall) Rehder], and black [*Aronia melanocarpa* (Michx.) Elliott] chokeberry species were tested for their total phenols and antioxidant activity and growth inhibitory activity against HT-29 human colon cancer cells. The results showed that only the extract of black chokeberry (Aronia berries) was active toward HT-29 cells, and this activity correlated with its total phenolic content, antioxidant activity, and levels of caffeic and chlorogenic acids [18]. This was supported by another study, which showed that the anthocyanin-enriched blackberry extract possessed antioxidant and anti-inflammatory activities and antiproliferative property against HT-29 cells [19]. Interestingly, the anthocyanin-rich extract of Aronia berries was selectively cytotoxic toward HT-29 cells but not human NCM460 normal colon cells [20]. Additionally, Caco-2 human colon cancer cell proliferation was inhibited when exposed to the Aronia juice [21].

Mechanistically, Aronia berries suppress HT-29 cell growth by dual blockage at the G1/G0 and G2/M phases of the cell cycle through upregulation of cyclin-dependent kinase inhibitors (CDKIs) and downregulation of cyclin A and cyclin B1 [20]. They inhibit Caco-2 cell growth by causing G_2_/M cell cycle arrest through upregulation of the tumor suppressor carcinoembryonic antigen-related cell adhesion molecule 1 (CEACAM1) [21]. Anthocyanins present in a methanol extract of Aronia berries were found to inhibit inflammatory cytokines, the expression of solute carrier family 1 member 5 (SLC1A5), and the phosphorylation of mammalian target of rapamycin (mTOR) and its downstream targets in Caco-2 cells. Of these, SLC1A5 is an important amino acid carrier highly expressed in cancer cells to regulate cell proliferation and invasion. Hence, inhibition of this protein by an extract of Aronia berries implies some promise for the discovery of novel anticancer agents from these berries by targeting SLC1A5 [22]. In addition, anthocyanins of Aronia berries were found to inhibit Caco-2 cell growth through the Wnt/β-catenin signaling pathway [23]. 

Interestingly, the berry anthocyanidins exhibited selective cell growth inhibitory activity against A549 and H1299 human non-small-cell lung cancer (NSCLC) cells, and an equimolar combination of these compounds showed synergistic activity in cell proliferation, invasion, and migration. These types of activities have been proposed to result from their effects on the oncogenic Notch and Wnt signaling pathways and their downstream targets [24]. Cell migration is involved critically in angiogenesis and wound healing, for which GTPases, as hydrolase enzymes, bind to the nucleotide guanosine triphosphate (GTP) that controls cytoskeleton conformation and alters cell motility, in relation to the PI3K/Akt pathway [25]. Thus, berry-derived anthocyanidins could show some therapeutic potential for the targeted treatment of NSCLC and other cancers and for the prevention of cancer recurrence and metastasis [24,25,26].

It is well known that cancer stem cells (CSCs) are responsible for tumor initiation, development, metastasis, and resistance to radiotherapy and chemotherapy. Aronia juice was found to inhibit selectively the proliferation of P19 mouse embryonal carcinoma stem cells through upregulation of tumor suppressors p53 and p73 and downregulation of the antiapoptotic protein UHRF1 and the stemness factor Oct-4 [27]. This indicates that phenolic compounds of Aronia berries may inhibit the resistance of cancer cells to cancer therapies to potentiate the effectiveness of other anticancer agents. Thus, the cytotoxicity of gemcitabine against AsPC-1 human pancreatic cancer cells was found to be enhanced by the phenolic compounds present in Aronia berries [28].

It has been well demonstrated that the major phenolic compounds of Aronia berries show potential anticancer activity. Of these, the anticancer potential of cyanidin-3-*O*-galactoside has been well described, even though there are no clinical trial studies reported thus far. For example, the berry extracts containing cyanidin-3-*O*-galactoside as a predominant component were found to inhibit BGC-803 human gastric cancer cell growth through induction of cell apoptosis by various gene changes, including increases in Bax and Bak expression and decreases in Bcl-2 and Bcl-xl expression [29]. The small-molecule phenolic acid, chlorogenic acid, acts on p53 and related proteins, p38 mitogen-activated protein kinase (p38 MAPK), c-Jun amino-terminal kinase (JNK), c-Myc, ROS, and on other targets to inhibit the proliferation, migration, and invasion of cancer cells [30].

Liver and lung tumor growth was inhibited significantly when male NOD/SCID mice (18–22 g) were inoculated by human Huh7 hepatoma or H446 lung cancer cells and treated intraperitoneally (i.p.) with chlorogenic acid (25 mg/kg, daily) for 30 days after the tumors reached 100 mm^3^. No obvious toxicity was observed in mice even at a high dose (>200 mg/kg, i.p.) [31]. Mechanistically, chlorogenic acid inhibited hepatocellular carcinoma growth by down-regulating DNA methyltransferase 1 (DNMT1) that assists with DNA methylation, the most prevalent epigenetic modification [32]. Interestingly, chlorogenic acid was also found to show a synergistic effect with the anticancer drug, doxorubicin, against human U2OS and MG-63 osteosarcoma cells [33].

A phase I trial of chlorogenic acid in patients with advanced cancer with no effective treatment has been posted (NCT02136342, sponsor: Chinese Academy of Medical Sciences). This trial started on 13 May 2014 but terminated on 21 October 2014 (https://www.clinicaltrials.gov/ct2/show/NCT02136342?cond=chlorogenic+acid&draw=4&rank=6, accessed on 5 September 2022). Another phase I study concerning the tolerance and pharmacokinetics of an injection of chlorogenic acid for the treatment of advanced cancer started in September 2014 and was completed in October 2016 (NCT02245204, sponsor: Sichuan J.Z. Bio-chemical Science and Technology Development Co., Ltd., Chengdu, China) (https://www.clinicaltrials.gov/ct2/show/NCT02245204?cond=chlorogenic+acid&draw=2&rank=7, accessed on 5 September 2022). Following these, a trial on single arm, open-label, multicenter, phase Ib/IIa studies of chlorogenic acid for injection for safety and efficacy of advanced lung cancer patients was posted on 23 November 2018 (NCT03751592, sponsor: Sichuan J.Z. Bio-chemical Science and Technology Development Co., Ltd., Chengdu, China) (https://www.clinicaltrials.gov/ct2/show/NCT03751592?cond=chlorogenic+acid&draw=2&rank=9, accessed on 5 September 2022). These clinical trials indicate the anticancer potential of chlorogenic acid.

Quercetin has been documented in terms of its potential antitumor activity. It suppresses cancer cell growth through induction of apoptosis and autophagy by targeting the PI3K/Akt/mTOR, Wnt/β-catenin, and MAPK/ERK1/2 pathways, which are involved in tumor metabolism and mitochondrial function [34]. It also inhibits GLUT1-, 3-, and 4-mediated 2-deoxy-glucose transport to show potential anticancer activity. Thus, quercetin could be a promising compound lead for the development of anticancer agents by targeting tumor metabolites [35].

**Figure 2:**
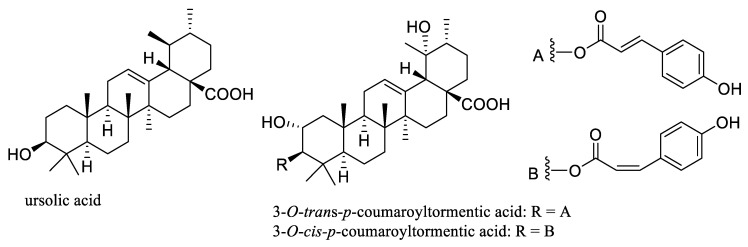
Structures of the triterpene ursolic acid and its derivatives, 3-*O*-*trans*- and 3-*O*-*cis*-*p*-coumaroyltormentic acids isolated from Aronia berries.

In addition, the pentacyclic triterpene ursolic acid that has been isolated from Aronia berries [36,37] (Figure 2) has shown potential anticancer activity through inhibiting NF-κB activation and other mechanisms involving angiogenesis and metastasis. A phase I study to assess the multiple-dose tolerability, efficacy, and pharmacokinetics of a liposomal form of ursolic acid indicated that this agent is safe and well-tolerated, and it showed some potential for improving patient remission rates [38,39]. Additionally, ursolic acid exhibited anti-inflammatory activity by targeting histamine, lipoxygenase, cyclooxygenase, phospholipase, nitric oxide, and ROS, with all of these found to play an important role in mediating potential antitumor activity of this triterpenoid [40,41].

Recently, two ester derivatives of ursolic acid, namely, 3-*O*-*trans*- and -*cis*-*p*-coumaroyltormentic acids (Figure 2), were identified as the active compounds of an ethyl acetate-soluble extract of Aronia berries by an activity-guided isolation procedure [42]. These esters were found to inhibit MCF-7 and MDA-MB-231 human breast cancer cell proliferation and mammosphere formation through deregulation of the expression of c-Myc, a cancer stem cell survival factor. The results obtained indicate that these triterpene esters could exert inhibitory activity against breast cancer stem cells, and thus they may be promising leads for the development of new breast cancer chemotherapeutic agents via disruption of c-Myc protein [42].

Hence, the abundance of potent antioxidant phenolic compounds and triterpenoid constituents support the anticancer potential of Aronia berries, which could be mediated by some unusual mechanisms of action. As an example, the levels of oxidative/nitrative stress and hemostatic activity in plasma collected from breast cancer patients were reduced significantly when the blood samples were treated with a commercial extract of Aronia berries (50 μg/mL) in vitro. This indicates that Aronia berries may represent a promising antioxidant therapy or co-therapy for breast cancer patients [43]. Thus, Aronia berries and their constituents could be promising leads for the development of new anticancer agents, as indicated by the cancer clinical trial investigations for chlorogenic acid in particular, a major phenolic acid of Aronia berries, as discussed above.

## 4. Potential Anti-Infective Activity of Aronia Berries

As documented in previous studies, Aronia berries show potent inhibitory activity toward various types of infections, which have been attributed to their potent antioxidant components [1,2,3]. For example, both aqueous and ethanolic extracts of Aronia berries were found to exhibit antimicrobial activity against foodborne strains of *Escherichia coli*, *Staphylococcus aureus*, and *Streptococcus pyogenes* [44], and their individual phenolic compounds showed different antimicrobial effects [45]. An in vivo study showed that the oxidative stress factors and other inflammatory cytokines were attenuated when six-week-old ICR male mice ingested a 3% (*w*/*v*) solution of dextran sulfate sodium (DSS) for seven days and were treated orally with an ethanol extract of Aronia berries (100, 300, or 600 mg/kg) plus drinking DSS/water for 29 days. These observations indicate that Aronia berries may possess some therapeutic potential against inflammatory bowel disease [46]. Additionally, an anthocyanin-enriched extract of Aronia berries exhibited anti-inflammatory activity by inhibiting NF-κB [47]. This type of extract was also found to destroy the integrity of the cell wall and membrane, to hinder protein synthesis, to induce protein degradation, and to inhibit DNA replication, transcription and expression, to suppress the growth of *Escherichia coli* [48]. Moreover, both cyanidin-3-*O*-galactoside and ursolic acid show potential anti-infective properties [29,39,49]. In a pilot study, the incidence of urinary tract infection and the use of antibiotics were found to be reduced when elderly residents of nursing homes consumed Aronia juice regularly for six months [50]. 

**Figure 3:**
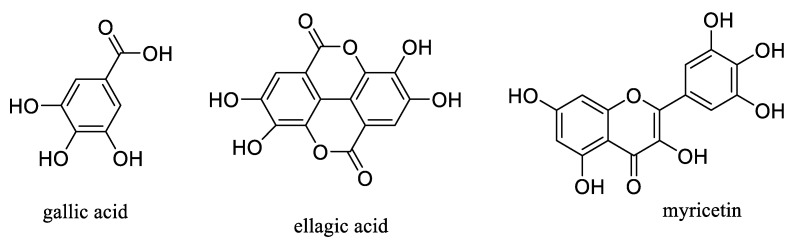
Structures of gallic acid, ellagic acid, and myricetin identified from Aronia berries.

Aronia berries contain considerable amounts of bioactive compounds, of which anthocyanins, proanthocyanidins, and other types of flavonoids and phenolic acids present all show antiviral activity against influenza viruses. These components inhibit virus replication directly and indirectly, including by blocking viral surface glycoproteins and by stimulating the immune system of the organism. Thus, Aronia berries could have potential use in the prevention and treatment of influenza, a dangerous disease with a risk of complications [51]. For example, a powder of Aronia berries was found to show anti-influenza activity, which could be attributed to their phenolic constituents, ellagic acid and myricetin (Figure 3) [52].

The combined or individual aqueous extracts of the frozen fresh fruits of *Aronia melanocarpa* and *Sambucus nigra* L. (elderberries) were tested against four human respiratory tract viruses, influenza A virus (A/H1N1), β-coronavirus-1 (HCoV-OC43), human herpesvirus type 1 (HHV-1), and human adenovirus type 5 (HAdV-5), of which A/H1N1 and HCoV-OC43 belong to the same β-coronavirus group as the virus found to be the cause of the current pandemic, COVID-19. A combined extract of these two types of berries showed antiviral activity against A/H1N1 and HCoV-OC43, but their activity toward HHV-1 and HAdV-5 was much less evident. The individual extracts of Aronia berries or elderberries exhibited inhibitory activity against A/H1N1, but neither of these extracts possessed any activity against HCoV-OC43 [53]. These results indicate that the mechanisms of action of the extracts of Aronia berries or elderberries against A/H1N1 and HCoV-OC43 could be different, and that the components of these berries may synergize with each other in their inhibitory effects against HCoV-OC43.

Viral infections are associated with oxidative stress, which leads to virus replication in infected cells. In addition, a coronaviral infection could activate the innate and acquired immune systems and increase the production of free radicals, and thus antioxidants could be essential in eliminating free radicals and in boosting the immune system to inhibit viral infections [54]. It has been well demonstrated that Aronia berries and their phenolic components show potent antioxidant activity [8]. They may also possess immunomodulating and anti-inflammatory activities, as indicated by their inhibition of the lipopolysaccharide (LPS)-induced nitric oxide (NO) production in RAW264.7 murine macrophage cells [55]. This has been also supported by several in vivo investigations. When two-month-old BALB/*c* mice were treated orally with an ethanol extract of Aronia berries (50 mg/kg, daily) for seven days, the proportion of CD11c^+^ dendritic cells in the mouse gut-associated lymphoid tissue (GALT) inclusive of Peyer’s patches (PP) and mesenteric lymph nodes (MLN) was increased significantly. However, the proportion of CD11b^+^ macrophages, CD8^+^ cytotoxic T lymphocytes, or CD4^+^ T helper lymphocytes did not change. Additionally, in mouse spleens, the proportion of CD4^+^ was downregulated, but no changes were observed in those of CD11b^+^, CD11c^+^, or CD8^+^. Importantly, a successful eradication of *Listeria monocytogenes* was observed when two-month-old BALB/*c* mice were treated orally with an ethanol extract of Aronia berries (50 mg/kg, daily) for seven days followed by infection with *L. monocytogenes*. Interestingly, in the GALT of the infected mice, the proportion of CD11b^+^ or CD8^+^ was increased, with no changes observed in those of CD11c^+^ or CD4^+^. Moreover, in the spleens of the infected mice, the proportion of macrophages or CD8^+^ was up-regulated, but that of CD11c^+^ was decreased, with no changes being observed in the proportion CD4^+^ [56].

The antioxidant phenolic compounds and triterpenoids of Aronia berries have also been well documented for their potential activity against severe acute respiratory syndrome coronavirus type 2 (SARS-CoV-2). For example, the anti-COVID-19 potential of ursolic acid has been described recently, based on its specific molecular targets and signaling pathways [57]. In turn, the anti-SARS-CoV-2 activity of quercetin has been evaluated in several COVID-19 clinical trials. 

To evaluate the benefits of quercetin in the prevention of COVID-19, a “Randomized, placebo-controlled clinical trial to evaluate the efficacy of an oral nutritional supplement based on quercetin in the prevention of COVID-19 infection for a duration of three months” started on 12 January 2021 and was completed on 25 May 2021 (NCT05037240, sponsor: Azienda di Servizi alla Persona di Pavia) (https://www.clinicaltrials.gov/ct2/show/NCT05037240?cond=quercetin&draw=4&rank=21, accessed on 9 September 2022). Another such trial, namely, “Study to investigate the benefits of dietary supplement quercetin for early symptoms of COVID-19” was initiated on 11 January 2021 and completed on 29 August 2021 (NCT04861298, sponsor: King Edward Medical University) (https://www.clinicaltrials.gov/ct2/show/NCT04861298?cond=quercetin&draw=4&rank=26, accessed on 9 September 2022).

To evaluate the COVID-19 therapeutic effects of quercetin, a clinical trial for the “Possible effect of quercetin on prophylaxis and treatment of COVID-19” was initiated on 20 March 2020 and completed on 31 August 2020 (NCT04377789, sponsor: Kanuni Sultan Suleyman Training and Research Hospital) (https://www.clinicaltrials.gov/ct2/show/NCT04377789?cond=quercetin&draw=3&rank=20, accessed on 9 September 2022). Another early phase I trial for “Effectiveness of quercetin in the treatment of SARS-CoV-2” was posted on 1 June 2021 (NCT04853199, recruiting, sponsor: Hôpital Universitaire Sahloul) (https://www.clinicaltrials.gov/ct2/show/NCT04853199?cond=quercetin&draw=2&rank=10, accessed on 9 September 2022).

The effects on COVID-19 of quercetin when combined with other agents have also been evaluated in clinical trials. A clinical trial for “Treatment benefits of flavonoids quercetin and curcumin supplements for mild symptoms of COVID-19” was initiated on 25 October 2021 and completed on 31 December 2021 (NCT05130671, sponsor: King Edward Medical University) (https://www.clinicaltrials.gov/ct2/show/NCT05130671?cond=quercetin&draw=4&rank=24, accessed on 9 September 2022). Another trial for “Complementary therapy of dietary supplements curcumin, quercetin, and vitamin D3 for mild to moderate symptoms of COVID-19” was initiated on 16 August 2021 and completed on 28 February 2022 (NCT04603690, sponsor: Liaquat University of Medical & Health Sciences) (https://www.clinicaltrials.gov/ct2/show/NCT04603690?cond=quercetin&draw=4&rank=25, accessed on 9 September 2022). Additionally, a phase IV clinical trial for “The study of quadruple therapy zinc, quercetin, bromelain and vitamin C on the clinical outcomes of patients infected with COVID-19” was initiated on 20 June 2020, with the recruitment status being unknown (NCT04468139, sponsor: Ministry of Health, Saudi Arabia) (https://www.clinicaltrials.gov/ct2/show/NCT04468139?cond=quercetin&draw=4&rank=28, accessed on 9 September 2022). These clinical trial investigations indicate that quercetin, a major constituent of Aronia berries, may be an important lead compound for the development of new anti-COVID-19 agents. 

Antioxidants prevent the production of free radicals that damage the normal cells and thus support human health, since it is well known that oxidative stress plays an important role in the pathogenesis of various diseases. The abundant phenolic compounds present in Aronia berries exhibit potent antioxidant activities, and thus intake of these agents may lead to decreased infection progression.

## 5. Potential Benefits to the Prevention and Treatment of Cardiovascular Diseases of Aronia Berries

Aronia berries are a valuable source of antioxidant compounds that can upregulate endothelial nitric oxide (NO) synthase and decrease oxidative stress and inflammatory gene expression to decrease the levels of blood triacylglycerols, total cholesterol, and low-density lipoprotein. Thus, consumption of these fruits could improve the lipid balance in patients with metabolic syndrome to prevent cardiovascular disease [58]. A three-arm, crossover, randomized, double-blind, and placebo-controlled intervention study was conducted to examine the impact of Aronia berry juice on the transcriptome in peripheral blood mononuclear cells from 19 subjects at cardiovascular risk. The results indicate that the prolonged habitual consumption of phenol-rich Aronia juice may lead to immunomodulatory activity mediated by different biological pathways [59]. A parallel and placebo-controlled study was performed to determine the impact of a four-week period of daily consumption of phenol-rich Aronia juice on long interspersed nucleotide element-1 (*LINE-1*) DNA methylation in peripheral blood leukocytes and on plasma polyunsaturated fatty acids (PUFA) profiles in subjects with a risk of cardiovascular disease. The results showed that consumption of Aronia berries decreased *LINE-1* methylation levels and the arachidonic acid/eicosapentaenoic acid ratio. Cardiovascular disease is associated with alterations in DNA methylation and PUFA profile, and thus this clinical trial indicates some potential cardioprotective effects of the habitual consumption of Aronia juice [60].

A meta-analysis of controlled clinical trials showed that daily supplementation with an extract of Aronia berries for six–eight weeks reduced significantly the systolic blood pressure and total cholesterol of adult participants, the main factors for cardiovascular disease risk [61]. After a one-month supplementation with a commercial extract of Aronia berries in patients with metabolic syndrome, the dangerous risk factors for cardiovascular disease, the overall potential for coagulation, clot formation, and fibrinolysis, were found to be decreased significantly, with blood pressure, glycemia, and the lipid profile all being reduced [62,63]. In another clinical trial investigation, the average 24 h and awake systolic and diastolic blood pressure was decreased significantly when 23 patients with no pharmacologically treated grade I hypertension consumed daily 200 mL of a phenolic compound-rich Aronia juice for four weeks. Additionally, their triglyceride and total low-density lipoprotein cholesterol levels were reduced significantly after a four-week consumption of the Aronia juice. These results indicate that Aronia juice may prevent the occurrence of cardiovascular disease, and the phenolic compounds that possess potent antioxidant activity may be the main active components involved [64]. This indication has been supported by the following clinical trial investigation. A double-blind, placebo-controlled, parallel designed study was conducted in 66 healthy men who consumed either (poly)phenol-rich extract of Aronia berries or the whole fruit powder of Aronia berry for 12 weeks. Consumption of Aronia berries led to a significant increase in flow-mediated dilation (FMD), and associations between changes in FMD, the Aronia berry-derived phenolic metabolites, and specific gut microbial genera were observed. Thus, regular consumption of Aronia berries could support cardiovascular health in individuals at low risk of cardiovascular disease [65].

Berry-derived phenolic compounds target several signaling pathways pertinent to the development of cardiovascular disease, including inflammation, oxidative stress, and cardiac and vascular remodeling. Of these, the non-isoflavone phenolic compounds present can also bind to the estrogen receptor (ER) to act as potential phytoestrogens. Both the ER and androgen receptor (AR) are expressed throughout the cardiovascular system, and thus mediation of ER and AR can contribute to cardiovascular health and disease. Hence, the berry-derived phenolic compounds may facilitate a targeted therapy for cardiovascular diseases [66]. Of these, chlorogenic acid and quercetin, two of the major phenolic components of Aronia berries, have been evaluated in clinical trials for their benefits to the prevention and treatment of cardiovascular diseases.

To test the potential activity of chlorogenic acid against cardiovascular disease, a clinical trial titled “Effect of a natural supplement containing chlorogenic acid and luteolin on cardio-metabolic risk factors in patients with metabolic syndrome” has been completed (from 1 June 2017 to 28 February 2018, NCT03444558, sponsor: University of Palermo) (https://www.clinicaltrials.gov/ct2/show/NCT03444558?cond=chlorogenic+acid&draw=2&rank=8, accessed on 13 September 2022). Following this, another such study “Effect of chlorogenic acids on the human vasculature” has also been completed (from 14 March 2018 to 20 December 2018, NCT03520452, sponsor: Nestlé) (https://www.clinicaltrials.gov/ct2/show/NCT03520452?cond=chlorogenic+acid&draw=4&rank=2, accessed on 13 September 2022).

A phase II clinical trial investigation to test the anti-inflammatory and anti-senescence effects of quercetin on the coronary arteries was posted on 28 May 2021, which is still recruiting (NCT04907253, sponsor: Montreal Heart Institute) (https://www.clinicaltrials.gov/ct2/show/NCT04907253?cond=quercetin&draw=5&rank=34, accessed on 13 September 2022). A phase III clinical trial entitled “Serum concentration and gene expression of sirtuin-1 and advanced glycation end-products in postmenopausal women with atherosclerotic coronary disease after administration of atorvastatin and supplementation with quercetin: Randomized trial” was posted on 2 August 2019, with an unknown present recruitment status (NCT03943459, sponsor: InCor Heart Institute) (https://www.clinicaltrials.gov/ct2/show/NCT03943459?cond=quercetin&draw=5&rank=36, accessed on 13 September 2022). However, a phase IV clinical trial titled “Quercetin for cardio-skeletal muscle health and estrogen deficiency (quickened) feasibility study in older women” has been suspended, which started in September 2022 (NCT04258410, sponsor: Wake Forest University Health Sciences) (https://www.clinicaltrials.gov/ct2/show/NCT04258410?cond=quercetin&draw=13&rank=13, accessed on 13 September 2022).

These clinical trial investigations indicate that the active components of Aronia berries with potential effects on cardiovascular disease are the phenolic compounds present, which show potent antioxidant activity to decrease the risk of cardiovascular disease [58]. Of these phenolic substances, both chlorogenic acid and quercetin can be regarded as possible lead compounds for the development of new agents to treat and prevent cardiovascular disease. 

## 6. Potential Antidiabetic Activity of Aronia Berries

Aronia berries exhibit potential antidiabetic activity, which could be mediated by their ability to combat hyperglycemia-induced oxidative stress [67]. In a diabetic animal model, the levels of blood glucose and serum insulin and the degree of insulin resistance were reduced significantly, with the glucose tolerance level and hepatic glycogen being increased, when male Wistar rats (around 200 g) were gavaged daily with an ethanol extract of Aronia berries (100 mg/kg) for eight weeks [68]. In an open-label trial, 35 type 2 diabetic (T2D) patients consumed the phenol-rich Aronia juice (150 mL/three times/day, 50 mL/each time), along with their standard diabetic therapy. The health status of the patients was found to be improved, indicating that Aronia juice might be a promising agent for the prevention and treatment of diabetes mellitus [69].

Interestingly, Aronia berries have shown some beneficial effects in terms of the treatment of type 1 diabetes (T1D). Different from type 2 diabetes (T2D), due to the inefficient use of insulin, T1D is caused by an autoimmune reaction, for which pancreatic β cells are damaged, and insulin cannot be produced. Thus, T1D is insulin-dependent and associated with a serious organ failure, owing to high blood glucose levels. In a streptozotocin (STZ)-induced T1D animal model, the blood glucose levels were found to be decreased, with the mouse pancreas β cells being protected, when six-week-old ICR healthy male mice were injected by STZ (i.p., single dose, 80 mg/kg) and gavaged daily with an ethanol extract of Aronia berries (10 or 100 mg/kg) for 31 days. These results indicate that consumption of Aronia berries could be effective in protecting pancreatic β cells and in the treatment of T1D [70].

Aronia berries are rich in antioxidant phenolic compounds that show anti-inflammatory activity, which may be translated into potential preventive and therapeutic effects for metabolic disorder, as well as for cancer, diabetes, and cardiovascular, kidney, and liver diseases [71,72]. Phenolic natural products also regulate carbohydrate and lipid metabolism and blood sugar and reduce insulin resistance, oxidative stress, and inflammation, and thus they have attracted wide interest in the potential control and prevention of diabetes [73,74]. For example, ellagic acid (Figure 3), obtained as a constituent of Aronia berries [52], was found to mitigate hepatic oxidative stress and insulin resistance in a T2D animal model. The blood glucose and insulin resistance were reduced significantly when 11-month-old Goto-Kakizaki female rats were gavaged daily with ellagic acid (50 mg/kg) for 28 days. This implies an antidiabetic effect of this phenolic compound for the treatment of hepatic complications in T2D [75].

**Figure 4:**
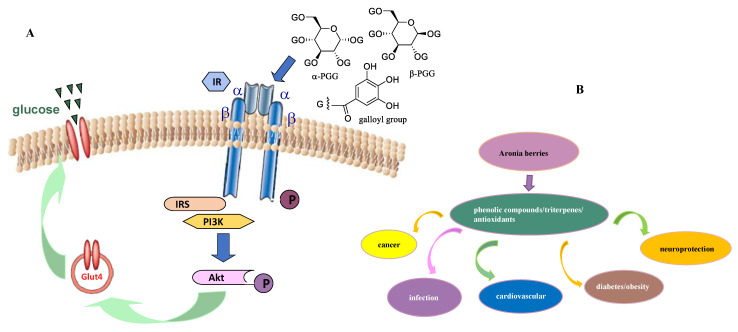
(**A**) The signaling pathway proposed for mediation of antidiabetic activity of α-PGG, in which α-PGG activates the insulin receptor (IR) in terms of its glucose transport stimulatory activity. It induces phosphorylation of the IR and Akt and activates PI3K followed by stimulating membrane translocation of GLUT 4 to keep glucose transport into cells. (**B**) Aronia berries and their potential beneficial activities in the promotion of human health.

In addition, β-pentagalloylglucoside (β-PGG), a penta-glucoside of gallic acid that is present in Aronia berries (Figure 3) [70], was found to exhibit potential antidiabetic activity, but its isomer, α-PGG, was more active. α-PGG targets the insulin receptor (IR) to activate the insulin-mediated glucose transport signaling pathway, including PI3K/Akt/GLUT4 (Figure 4A) [76,77]. The antidiabetic activity α-PGG was improved by introducing a chlorine atom at the C-6 position of the glucose core. The target compound produced, 6-chloro-6-deoxy-1,2,3,4-tetra-*O*-galloyl-α-D-glucopyranose (6Cl-TGQ), was more potent than its parent compound, α-PGG. It is efficacious orally using the T1D and T2D animal models and targets selectively the insulin receptor signaling pathway. Thus, 6Cl-TGQ could be a promising agent for the prevention and treatment of T1D and T2D [78]. Additionally, quercetin has been evaluated for its antidiabetic activity. A phase II clinical trial for “Inhibition of intestinal glucose absorption by the bioflavonoid quercetin in the obese and in obese type 2 diabetics” has been completed (from 30 April 2010 to 6 August 2021, NCT00065676, sponsor: National Institute of Diabetes and Digestive and Kidney Diseases) (https://www.clinicaltrials.gov/ct2/show/NCT00065676?cond=quercetin&draw=5&rank=31, accessed on 13 September 2022). Another phase II clinical trial for “Evaluation of quercetin in type 2 diabetes: Impact on glucose tolerance and postprandial endothelial function” has been completed (from May 2013 to March 2015, NCT01839344, sponsor: Bastyr University) (https://www.clinicaltrials.gov/ct2/show/NCT01839344?cond=quercetin&draw=3&rank=14, accessed on 13 September 2022).

Thus, as summarized above, Aronia berries show potential anti-T1D and anti-T2D activity, for which the phenolic constituents present seem to be the main contributors. These compounds mediate their antidiabetic properties by relieving hyperglycemia-induced oxidative stress. The antidiabetic potential of α- and β-PGG has been evaluated preclinically, and that of quercetin has been investigated in several clinical trials for the prevention and treatment of diabetes.

## 7. Miscellaneous Bioactivities of Aronia Berries

The phenolic compounds of Aronia berries may form stable complexes with toxic metals to prevent their absorption and uptake into tissues, and thus these compounds could protect humans from the toxic action of these metals [79]. As discussed previously, phenolic compounds may protect against cadmium-induced liver injury [80]. In addition, Aronia berries show protective effects on human health through other mechanisms. In an animal model, indomethacin-induced gastric mucosal damage accompanied by oxidative stress was found to be reduced dose-dependently and significantly, with an increased gastric mucus production observed, when male Wistar rats (200–250 g) were gavaged with Aronia juice (single dose, 5, 10 or 20 mL/kg) for one hour followed by subcutaneous treatment with indomethacin (30 mg/kg), and then examined biologically four hours after indomethacin induction [81].

Aronia berries also show evidence of having neuroprotective potential. An ethanol extract of Aronia berries was found to reduce intracellular ROS and Ca^2+^ levels to protect HT-22 mouse hippocampal neuronal cells from glutamate-induced death [82]. The learning ability and memory and brain morphology of aged rats were improved when 24-month-old healthy male Wistar rats were treated orally with Aronia juice (10 mL/kg, daily) for 105 days [83]. Following an activity-guided phytochemical investigation on Aronia berries, anthocyanins were identified as the major active components. These compounds exhibited potent antioxidant and neuroprotective effects against amyloid-β-induced cognitive impairment [84]. Additionally, chlorogenic acid has been discussed in a recent review article for its potential neuroprotective properties [85]. Ursolic acid exhibits multiple bioactivities, including antioxidative stress, antineuroinflammation, antiexcitotoxicity, mitochondrial protection, enzyme inhibition, and receptor modulation. Thus, it possesses therapeutic potential in neurodegenerative and neuropsychiatric diseases, including Alzheimer’s and Parkinson’s diseases, anxiety and depression, and multiple sclerosis [86].

Aronia berries also show some potential for body weight control and obesity [3]. Mouse body weight and food intake were reduced significantly when four-week-old male C57BL/6N mice were gavaged daily with a cyanidin-3-*O*-galactoside-enriched extract of Aronia berries (50 mg/kg) for eight weeks. Such a treatment also resulted in serum levels of leptin, insulin, triglyceride, total cholesterol, and low-density-lipoprotein (LDL) cholesterol being decreased. This indicates that the cyanidin-3-*O*-galactoside-enriched Aronia berry preparation may be useful for the treatment of obesity [87].

## 8. Concluding Remarks

Natural products offer a valuable source for the discovery of pharmaceutical agents to support human health, from which the novel structures produced have been long used for the design and discovery of new drug entities to treat effectively human diseases. Of these, plants play a key role in improving human health historically, and many effective plant-derived agents have been developed as effective agents for the treatment of cancer and other diseases over the past a few decades [88,89,90]. For example, a large number of plants have been used traditionally as medicinal herbs in the health care system of Laos and remain the backbone of the primary health care of this country [91]. Additionally, traditional herbal medicines represent an alternative in the treatment of animal diseases and some human conditions in Turkey [92].

Currently, cardiovascular diseases, infections, cancer, diabetes, obesity, and brain disorders are major problems that impair human health. All of these diseases involve oxidative stress that leads to cell apoptosis and the increased progression of their pathogenesis. Thus, antioxidants may be important in supporting human health, owing to their prevention of the production of free radicals that damage normal cells. Phenolic components of fruits and vegetables were found to show effects on human health through the antioxidant activity [93], of which dieckol, a phlorotannin identified from several brown algal species, was found to show promising effects on promotion of human health though its antioxidant potential [94]. It is well documented that antioxidants can help control infections, which, in turn, can be supportive of the treatment of other diseases [93]. As discussed previously and in the present review, Aronia berries show potent antioxidant activity and thus have been found to be potentially beneficial for the prevention and possible treatment of cancer, cardiovascular diseases, diabetes, obesity, and brain disorders [1,2,3,5,8] (Figure 4B). Hence, Aronia berries may provide an important strategy to be used in the promotion of human health.

This has been supported by several pilot studies and a meta-analysis for Aronia berries, which showed that regular consumption of Aronia juice can reduce the incidence of urinary tract infection and the use of antibiotics of elderly residents of nursing homes [50] and decrease blood pressure and total cholesterol levels to show potential cardioprotective effects [60,61,62,63,64,65]. In addition, Aronia berries were found to show immunomodulating activity [55,56,59], and they exhibited synergistic effects with elderberries for their inhibitory potency against β-coronavirus-1 (HCoV-OC43) [53]. Thus, the fruits of A. melanocarpa could be used with other agents to enhance their pharmaceutical properties in future development efforts. Importantly, Aronia berries are cultivated successfully in the U.S. and in other countries and will be available in sufficient supply for the possible development of pharmaceutical or health-promotion agents. However, although Aronia berries and their constituents have been shown to afford beneficial effects on the prevention and treatment of diseases associated with oxidative stress, their effectiveness as observed in clinical trial investigations to date seems to be poor [95]. Thus, future investigations for Aronia berries could focus more on optimizing the doses of phenolic and other constituents present and on new formulation development, as well as the isolation of new active compounds and their synthetic modifications. 

Both phenolic compounds and triterpenoids have been identified as the major antioxidant components of Aronia berries, which contribute to the majority of the other biological effects observed for these berries. Thus, these active components could be regarded as promising lead compounds in the future development of new therapeutic agents. This can be supported by the unique mechanisms observed for these compounds and the outcomes of several clinical trial investigations performed. For example, chlorogenic acid shows potential antitumor activity by inhibiting DNA methyltransferase 1 (DNMT1) expression [30,31,32,33]. Thus far, several cancer clinical trial investigations have been performed for this compound. Quercetin targets NF-κB and the PI3K/Akt/mTOR, Wnt/β-catenin, and MAPK/ERK1/2 pathways to exert its antitumor activity [34]. This compound has been evaluated for its therapeutic potential in several clinical trials for the prevention and treatment of cancer, cardiovascular disease, COVID-19, and diabetes. In addition, a major triterpene of Aronia berries, ursolic acid, has also been tested in cancer clinical trials [38,39]. Its ester derivatives, 3-*O*-*trans*- and -*cis*-*p*-coumaroyl-tormentic acids, derived also from Aronia berries, were found recently to target c-Myc to exert inhibitory activity against breast cancer stem cells. Thus, these triterpene esters appear to show some promising cancer therapeutic effects via disruption of the c-Myc protein [42]. Therefore, chlorogenic acid and ursolic acid can be used for the design of effective anticancer agents, and quercetin could be regarded as a promising lead compound for the control of COVID-19 or other coronoviral infections and cardiovascular diseases.

Although phenolic compounds have been identified as the major active components of Aronia berries, the antidiabetic, antiobesity, and neuroprotective components of these berries have not been determined unequivocally. Ursolic acid and its derivatives, as well other constituents of Aronia berries showing potential antitumor activity still need to be investigated further. Additionally, it is possible that some new compounds that show anti-infective activity and benefits in the treatment of cardiovascular diseases could be identified from Aronia berries.

**Figure 5:**
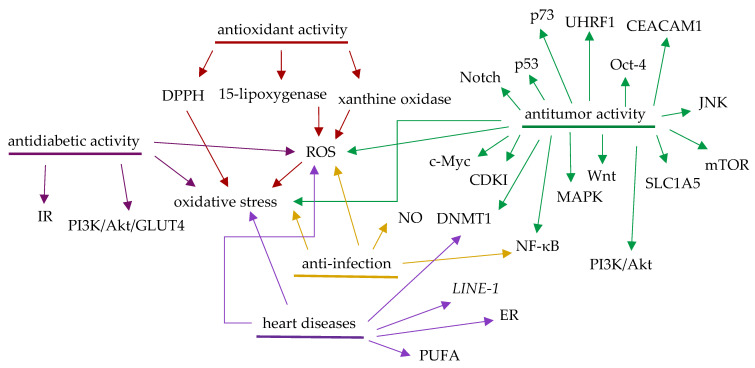
Molecular targets identified for mediation of pharmaceutical properties of Aronia berries.

It is worthy of note that Aronia berries were found to show potential anti-influenza activity [51,52], and one of its major components, ursolic acid, exhibited anti-COVID-19 activity [57], while another major compound, quercetin, has been evaluated in several clinical trials for the prevention and treatment of COVID-19. Ursolic acid also showed potent NF-κB inhibitory activity, indicating that this triterpene may mediate its bioactivity by targeting NF-κB [38,96]. NF-κB is a major protein involved in cancer and infection and could be an important linker between these conditions. Inflammation is an immediate body response to tissue injury caused by infection or other noxious stimuli, and persistent local inflammation perturbs the homeostatic control of cell signaling pathways to predispose cells to premalignant and malignant conversion. Of this, one mechanism is the ROS induction, which triggers Akt and activates NF-κB. While a growing tumor is surrounded by inflammatory immune cells, which will form an inflammatory tumor microenvironment (TME) and release growth factors, cytokines, and prostaglandins to stimulate proliferation, invasion, and metastasis of the preneoplastic cells [97]. Communication between the stroma and malignant cells enables cancer cells to invade normal adjacent tissues, indicating that the TME could contribute critically to the treatment of cancer. Thus, any agents showing anti-intratumoral inflammation activity could well inhibit tumor growth [98]. As shown in Figure 5, Aronia berries target oxidative stress to exhibit their potential therapeutic effects on cancer, cardiovascular diseases, diabetes, infections, and other diseases. In addition, these berries mediate their potential antitumor activity by targeting many proteins, including NF-κB, which is also involved in mediation of their anti-infective activity. Thus, Aronia berries could contribute to the TME-targeted cancer treatment, and further investigation on these berries by targeting TME may support the discovery of some novel agents to treat cancer and/or other diseases effectively.

Although the antitumor molecular targets of Aronia berries have been well investigated, the proteins identified for their other pharmaceutical properties are much less understood. Thus, more detailed mechanistic studies for the antidiabetic and anti-infective activities and potential therapeutic effects on heart disease of Aronia berries could support the development of these berries as useful agents in the promotion of human health.

## Data Availability

Not applicable.

## Sample Availability

Samples of the compounds are not available from the authors.
